# Heterologous protein-DNA interactions lead to biased allelic expression of circadian clock genes in interspecific hybrids

**DOI:** 10.1038/srep45087

**Published:** 2017-03-27

**Authors:** Danny W-K. Ng, Helen H. Y. Chen, Z. Jeffrey Chen

**Affiliations:** 1 Department of Molecular Biosciences and Center for Computational Biology and Bioinformatics, The University of Texas at Austin, Austin, Texas 78712, USA; 2 Department of Biology, Hong Kong Baptist University, Kowloon Tong, Hong Kong, China; 3 The Partner State Key Laboratory of Agrobiotechnology, The Chinese University of Hong Kong, Shatin, Hong Kong, China

## Abstract

Genomic interactions in allopolyploids create expression variation of homoeologous alleles through protein-protein and protein-DNA interactions. However, the molecular basis for this is largely unknown. Here we investigated the protein-protein and protein-DNA interactions among homoeologous transcription factors in the circadian-clock feedback loop, consisting of CCA1 HIKING EXPEDITION (CHE), CIRCADIAN CLOCK ASSOCIATED1 (CCA1), and TIMING OF CAB EXPRESSION1 (TOC1), plus the interaction with a chromatin factor, HISTONE DEACETYLASE1 (HD1). In the allotetraploids formed between *A. thaliana* (At) and *Arabidopsis arenosa* (Aa), *AtCCA1* is expressed at lower levels than *AaCCA1*, which could alter clock output traits. The reduced *AtCCA1* expressions in the allotetraploids are consistent with the biochemical data that AaCHE showed preferential binding to the *AtCCA1* promoter, in which AaCHE interacts with a higher affinity to AtHD1 than AtCHE. AaCHE also showed a higher affinity to TOC1 than AtCHE, consistent with the effect of TOC1 on repressing *CCA1*. Thus, stronger AaCHE-TOC1 and AaCHE-AtHD1 interactions reduce *AtCC1* allelic expression. Our current data suggest a biochemical basis for protein interactions *in trans* with a preference to the *cis*-acting elements in heterologous combinations to reduce *AtCCA1* expression, while altered *CCA1* expression has been shown to affect metabolic and biomass heterosis in interspecific hybrids or allotetraploids.

Heterosis refers to superior growth and fitness in the offspring relative to one or both parents. Since the discovery of the phenomenon by Charles Darwin in 1876^1^, heterosis has been widely applied in agriculture to improve the production of important crops such as maize and sorghum and farm animals such as pigs and sheep^2^,^3^. Compared to regular hybrids, heterozygotes and heterosis are permanently fixed in the allotetraploids such that mechanisms for molecular changes can be readily studied^4^,^5^. Although the genetic, genomic, and epigenetic bases of heterosis have been extensively studied^2^,^3^,^6^, biochemical bases for heterologous protein-protein and protein-DNA interactions in heterosis are poorly understood.

A recent study found that the heterozygote of *SFT* locus between an allele of the cultivated tomato and an introgressed allele from the wild tomato is associated with fruit yield heterosis^7^. This is consistent with the conventional example of hybrid (heterozygote) advantage for sickle cell anemia; individuals who are homozygous for the mutant allele have the disease, whereas heterozygous individuals have some susceptibility to the disease and are more tolerant to malaria infection than individuals who are homozygous for the normal allele^8^,^9^. This suggests heterozygotic (or overdominant) effect on heterosis^2^,^5^. Biochemical bases for this effect could be associated with heterodimeric interactions, leading to change in the activities or DNA-binding affinities of heteromers. For example, E2F is a mammalian transcription factor that binds directly to the adenovirus E2 promoter for activation^10^. Mixing individual E2F subunits together dramatically increases (100- to 1000-fold) the specific DNA binding activity. In maize, heterodimers of alcohol dehydrogenase are more stable than either homodimers, suggesting a biochemical basis for this case of single-locus heterosis^11^. Protein inhibitors of heterologous α-amylases and proteinases are useful for plant protection. In wheat, different subunits of these inhibitors, which are encoded by homoeologous genes on chromosomes 4A, 4B, and 4D, respectively, exist in hexaploid (AABBDD), tetraploid (AABB), and diploid (DD) wheats. Mixing subunits of the hexaploid wheat increases inhibitory activities to 133%, compared to mixing subunits of the diploid wheat (33%)^12^. Thus, heterodimers of protein and transcription factors are either more stable or have stronger binding activities to the promoters of downstream genes in various biological pathways, leading to heterosis^2^,^6^,^13^.

The heterozygote advantage model can be applied to other protein and transcription factors such as circadian clock regulators^5^,^6^,^14^. Circadian clocks regulate physiology and metabolism in plants and animals^15^,^16^,^17^,^18^. In *Arabidopsis*, the circadian clock mediates expression of 30% or more genes in any given growth conditions^19^,^20^. The central clock regulators consist of the morning regulators, CIRCADIAN CLOCK ASSOCIATED1 (CCA1) and LATE ELONGATED HYPOCOTYL (LHY), and the evening factor, TIMING OF CAB EXPRESSION1 (TOC1)^16^,^17^,^21^. CCA1 HIKING EXPEDITION (CHE), a TCP transcription factor, forms a negative feedback regulatory loop in mediating *CCA1* repression during the day^22^. TOC1 is a DNA-binding transcriptional repressor of *CCA1* at the evening phase^23^,^24^.

Interestingly, the morning-phased clock regulators are down-regulated, while the evening-phased ones are activated in *Arabidopsis* intraspecific hybrids^25^,^26^ and allotetraploids (doubled interspecific hybrids)^14^, which are formed by pollinating *Arabidopsis thaliana* with *Arabidopsis arenosa* pollen^27^. Specifically, both *A. thaliana CCA1 (AtCCA1*) and *A. arenosa CCA1 (AaCCA1*) are down-regulated in the allotetraploids during the day, which correlates with increased levels of chlorophyll and starch, promoting growth vigor^26^. Surprisingly, *AtCCA1* is expressed at lower levels than *AaCCA1* in the allotetraploids; the underlying mechanism is largely unknown. Here we take the advantage of the circadian transcriptional feedback loop to test a biochemical model for homoeologous protein-protein interactions and protein-DNA (*cis*-regulatory element) recognition in altering allelic expression levels. This biochemical model could explain a molecular basis for heterosis in interspecific hybrids and allotetraploids.

## Results

### *CHE* expression is affected by *hd1* mutation

**Figure 1:**
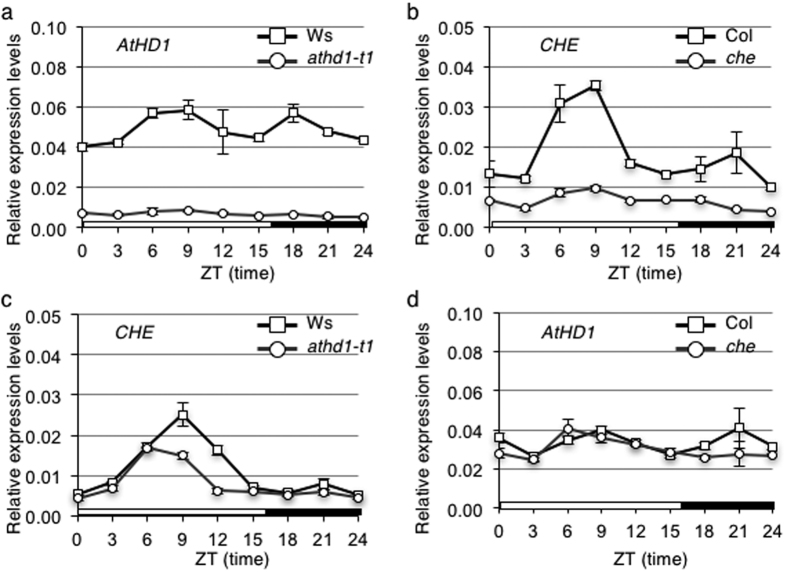
Repression of *CHE* in the *A. thaliana athd1* mutant. Quantitative real-time PCR showing temporal expression of *HD1* and *CHE* in the *A. thaliana* wild-type Ws or Col-0 (square) and its corresponding *athd1*-*t1* (in Ws, circle) or *che* mutant (in Col, circle). The target transcript levels were normalized with the *EF1α* endogenous control. ZT, Zeitgeber time; ZT0 = dawn. Open bar represents light period and fill bar represents dark period. (**a**) *HD1* expression in Ws and the *athd1* mutant. (**b**) *CHE* expression in Col and the *che* mutant. (**c**) *CHE* expression in Ws and the *athd1*-*t1* mutant. (**d**) *HD1* expression in Col and the *che* mutant. Error bars represent standard deviation from 3 replicates.

Chromatin modifications including histone acetylation and deacetylation mediate expression of circadian clock genes in mammals and plants^14^,^28^,^29^. In *A. thaliana*, histone deacetylation represses expression of circadian clock genes^29^,^30^. However, the genetic interaction between histone deacetylase and clock gene expression is unknown. *A. thaliana* Histone Deacetyase1 (AtHD1) is a general transcription repressor or activator^31^,^32^. In hybrid rice, expression of the rice histone deacetylase (*OsHDT1*) showed a circadian rhythm^33^. Here we tested if the *AtHD1* affects clock gene expression and *vice versa*, if the circadian clock affects *AtHD1* expression. In the wild-type plants, expression of *AtHD1* showed slight diurnal rhythms with relatively higher expression levels at ZT9 and ZT18 (ZT, Zeitgeber time; ZT0 = dawn) (Fig. 1a). As expected, *AtHD1* expression was abolished in the *athd1*-*t1* mutant (Fig. 1a). *athd1*-*t1* is a T-DNA insertion line in exon 2 of *AtHD1*, resulting in a null mutation^31^. In *Arabidopsis*, CHE was found to bind to the *CCA1* promoter and repress the promoter activities^22^. Using qRT-PCR, we have confirmed that CHE showed high expression peaks around ZT6 and ZT9 in plants grown under 16 h light/8 h dark cycles (Fig. 1b). This is consistent with the reported *CHE* expression to which it peaked at 9 hours after the subjective dark period under continuous light^22^. In the *che* mutant, this oscillated expression was lost in the *che* mutant. In the *athd1*-*t1* mutant^31^, *CHE* expression levels were reduced at ZT9 and ZT12 (Fig. 1c). In contrast, expression levels of *AtHD1* remained unchanged in the *che* mutant (Fig. 1d). These data suggest that *AtHD1* positively mediates *CHE* expression directly or indirectly. In contrast, CHE does not affect *AtHD1* expression.

### Roles of HD1, CHE and TOC1 in *CCA1* expression

**Figure 2:**
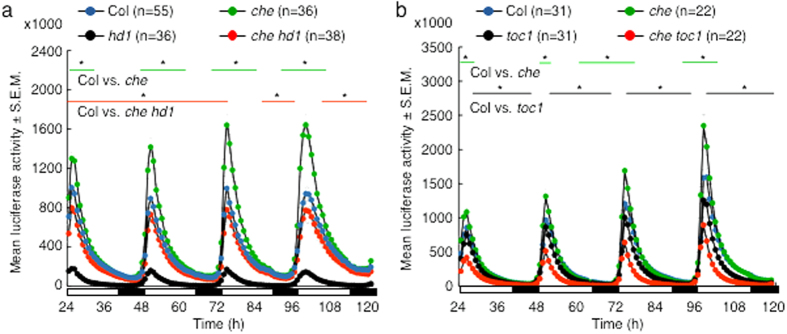
*ProCCA1:LUC* expression in *che, hd1* and *toc1* mutants. Bioluminescence counts (Mean ± S.E.M.; in thousands) from *ProCCA1:LUC* expression were measured in (**a**) wild type Col, the *che, hd1* and *che hd1* mutants; and (**b**) *toc1* and *che toc1* mutants, respectively. Measurements were taken with 8-days old seedlings over a 4-days period under 16 h light (open bars) and 8 h dark (filled bars) conditions. Lines with asterisks mark the range of time points with statistically significant differences between Col and the indicated mutant (P < 0.05, Student’s *t* test). For *hd1* or *che toc1, ProCCA1:LUC* expression was significantly down-regulated (P < 0.05) when compared to that in any other genotypes at all time points. Significant reduction (P < 0.05) of *ProCCA1:LUC* expression was also found in *che* and *che hd1* comparison. S.E.M. = Standard error of the mean.

Within the central loop of the circadian clock, CHE represses *CCA1* expression during the day^22^. In addition, using a *CCA1* promoter-driven luciferase (LUC) expression in bioluminescence analysis, Pruneda-Paz *et al*.^22^ revealed that the *CCA1* promoter activity was upregulated in the *che* mutant and down-regulated in 35S-driven CHE overexpressors^22^. Since *CHE* expression is affected in the *athd1*-*t1* mutant, HD1 and CHE could act cooperatively to repress *CCA1* expression. We tested this using a *ProCCA1:LUC* reporter line^34^ in the *che* or *hd1* single or the *che hd1* double mutant (Fig. 2a). Consistent with the previous finding^22^, CHE represses *CCA1*, and the *CCA1* promoter activity was upregulated in the *che* mutant (Fig. 2a). In the *hd1* mutant, *CCA1* is diurnally expressed^35^. Although oscillation of the *CCA1* promoter activity was observed, its peaks were dramatically reduced in the *athd1*-*1* mutant, indicating that HD1 acts an activator for *CCA1* expression. This is probably because expressions of other clock regulators, such as CHE and TOC1, are affected in the *athd1* mutant^30^,^36^. Indeed, down-regulation of the *CCA1* promoter activity was largely relieved in the *che hd1* double mutant, suggesting that CHE and HD1 act antagonistically in gating *CCA1* expression. TOC1 is an evening-expressed protein and it has long been recognized as a positive regulator of *CCA1*^37^,^38^,^39^. In the *toc1* mutant, *CCA1* expression peak was reduced compared to the wild type plants in qRT-PCR assays^38^,^39^. Consistent with data from these studies, expression of *LUC* driven by the *CCA1* promoter in the *toc1* mutant was down-regulated in the *ProCCA1:LUC* reporter line (Fig. 2b). While the activity of the *CCA1* promoter was upregulated in the *che* mutant, the *LUC* expression peaks were further reduced in the *che toc1* double mutant. Therefore, these data suggested a role for genetic interactions between TOC1 and CHE in regulating *CCA1* expression. Recent biochemical studies revealed that TOC1 indeed functions as a repressor of *CCA1*^23^,^24^. Further studies are needed to resolve the biochemical interactions among TOC1, CHE, and CCA1.

### Preferential binding of *A. arenosa* CHE homolog to the *A. thaliana CCA1* promoter

**Figure 3:**
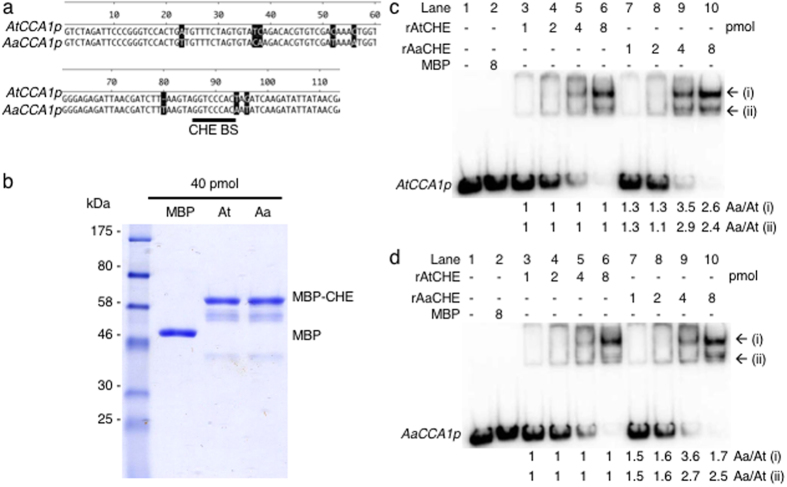
AaCHE preferentially bound to the *CCA1* promoter (*CCA1p*). (**a**) Sequence alignment of the *AtCCA1* promoter (*AtCCA1p*) (from −315 to −182 relative to the transcription start site, +1) and the *AaCCA1p* (from −325 to −191) fragments used in gel electrophoretic mobility shift assay (EMSA). The CHE binding site (CHE BS) is indicated. (**b**) Coomassie stain of purified recombinant AtCHE and AaCHE with N-terminus MBP fusion. MBP: maltose binding protein. (**c**,**d**) EMSA showing preferential binding of the recombinant AaCHE (rAaCHE) to either (**c**) *AtCCA1p* or (**d**) *AaCCA1p*. MBP alone is used as a negative control in the binding reaction. Shifted DNA bands (i and ii) caused by rAaCHE binding were quantified by imageJ densitometry and normalized against the corresponding shifted band caused by rAtCHE. The relative quantifications were indicated at the bottom.

In interspecific hybrids of animals and allotetraploids of plants^40^,^41^, *cis*- and *trans*-acting effects mediate expression of parental alleles originating from different progenitors. In *Arabidopsis* allotetraploids that are formed between *A. thaliana* (At) and *A. arenosa* (Aa), *AtCCA1* is expressed at lower levels than *AaCCA1*^14^ (see Supplementary Fig. S1a). The altered *CCA1* expression in the allotetraploids are consistent with the upregulation of morning-phased genes in photosynthetic and starch biosynthetic pathways^41^ that promote biomass heterosis^14^. The molecular basis for this differential *CCA1* allelic accumulation is unknown. We predict that this allelic *CCA1* repression results from changes in the *cis*-regulatory elements (promoters) of *CCA1* in combination of *trans*-acting effects by factors such as CHE and HD1 on *CCA1* expression^6^. In *A. thaliana*, CHE binds to the CHE-binding site of the *CCA1* promoter^22^,^34^ and represses *CCA1* expression. Although the *A. arenosa CCA1* promoter contains the same CHE-binding site, the flanking sequences were different compared to the *A. thaliana* promoter (Fig. 3a). The *cis*-element variation between *AtCCA1* and *AaCCA1* promoters could cause different binding activities by AtCHE and/or AaCHE, leading to allelic *CCA1* expression changes in allotetraploids (see Supplementary Fig. S1a). To test this, we cloned cDNA of AtCHE and AaCHE to produce recombinant proteins in bacterial cells (see Supplementary Fig. S2) and purified the proteins using a maltose binding protein (MBP) that was fused to the 5′ of the CHE protein. The quantity and quality of proteins were verified by Bradford assay and 10% SDS-PAGE, respectively (Fig. 3b). In a gel electrophoretic mobility shift assay (EMSA), the same amount of the purified recombinant proteins was used to test their binding affinities to the native *AtCCA1* or *AaCCA1* promoter (*AtCCA1p* or *AaCCA1p*) fragment containing the CHE binding site (Fig. 3a). The recombinant AaCHE (rAaCHE) had a 1-3-fold higher binding affinity than rAtCHE to the *AtCCA1p* promoter (Fig. 3c, compare lanes 5 with 9 and 6 with 10) than *AaCCA1p* promoter (Fig. 3d, compare lanes 5 with 9 and 6 with 10). These data suggest that rAaCHE preferentially bound to the *CCA1* promoter compared to rAtCHE.

**Figure 4:**
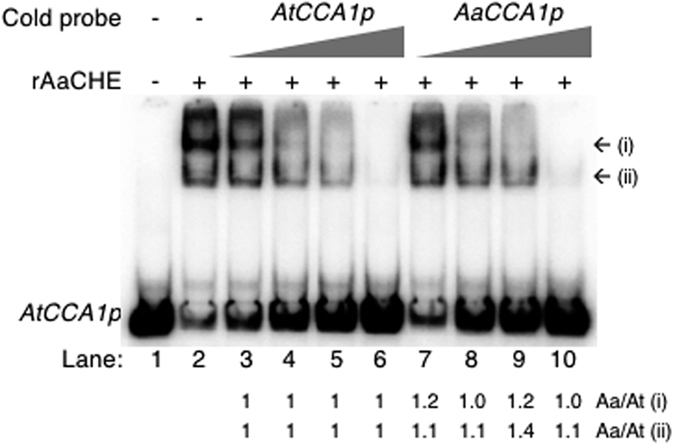
AaCHE preferentially bound to the *AtCCA1p*. Recombinant AaCHE (rAaCHE, 4pmole) was used for EMSA in the presence of ^32^P-labeled *AtCCA1p* (15 fmol) probe (lane 2). Competition assays were performed using 1x, 5x, 25x, and 125x of cold *AtCCA1p* (lanes 3–6) or *AaCCA1p* (lanes 7–10) probe as indicated. MBP (4 pmol) is used as a negative control (lane 1). The relative imageJ densitometry quantifications of shifted DNA bands (i and ii) in the presence of cold *AaCCA1p* (lanes 7–10; relative to the corresponding band in the presence of cold *AtCCA1p* in lanes 3–6) were indicated at the bottom.

In a competition assay using an increased amount of cold (unlabeled) *AtCCA1p* or *AaCCA1p* probe, CHE binding had a higher specificity to the *AtCCA1* promoter than to the *AaCCA1* promoter (Fig. 4). The cold *AaCCA1p* probe competitor caused more abundant mobility shift of the labeled *AtCCA1p* probe than the cold *AtCCA1p* probe competitor (compare lanes 5 with 9). Moreover, for the same amount of cold competitors, the cold *AtCCA1p* showed a higher affinity to rAaCHE than the cold *AaCCA1p* in competition with the labeled *AtCCA1p*. Therefore, these *in vitro* data suggest preferential binding of AaCHE to the *AtCCA1* promoter, which agreed with and could explain the lower expression level of *AtCCA1* than that of *AaCCA1* homoeolog in the allotetraploids *in vivo*^14^.

### Interactions between homoeologous CHE and HD1 proteins

HD1 is a general transcriptional repressor^36^,^42^, which could be recruited by CHE to repress target genes such as *CCA1*. It is unclear if CHE interacts with HD1 biochemically. Here we tested their protein-protein interactions through two approaches. A yeast two-hybrid assay has shown positive interactions between CHE and HD1 (see Supplementary Fig. S4a). Using AtCHE and AaCHE as baits, weak interactions were detected with the respective AtHD1 and AaHD1. Interestingly, by substituting AtHD1 with AaHD1 as prey when using AtCHE as bait, there was a slight increase in the interactions (AtCHE-AaHD1). Similarly, by substituting AaHD1 with AtHD1 as prey when using AaCHE as bait, the interaction level was increased. Overall, there was a trend of enhanced interactions between the proteins that originated in different species (AtCHE-AaHD1 or AaCHE-AtHD1) than that from the same-species (AtCHE-AtHD1 or AaCHE-AaHD1).

**Figure 5:**
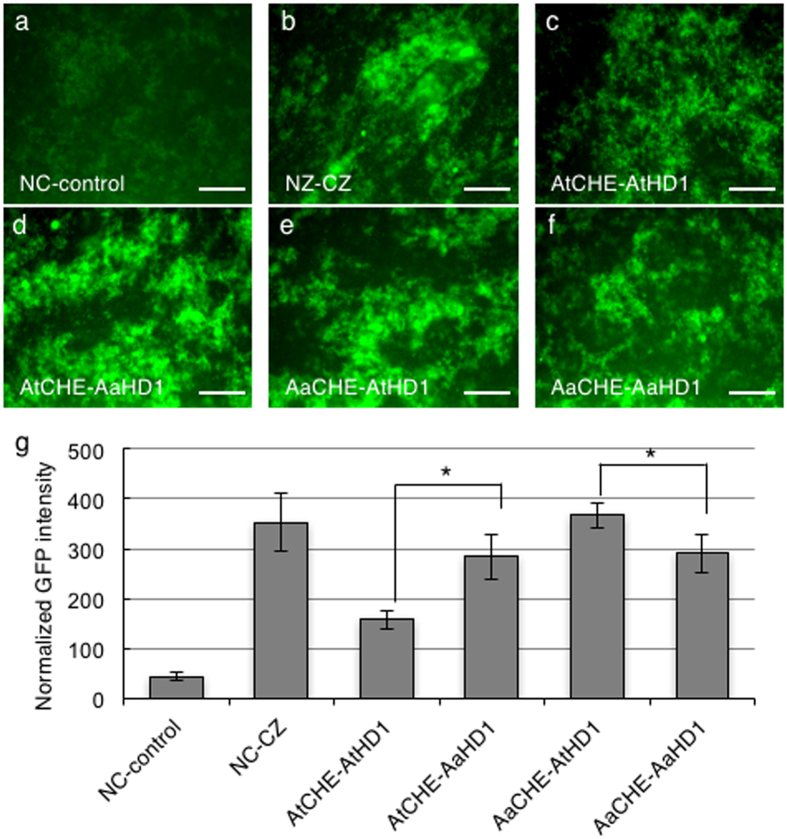
Protein-protein interactions between HDAC and CHE. Split GFP constructs were expressed in *E. coli* for quantification of protein-protein interaction. In the split constructs, the N-GFP was fused with CHE and the C-GFP was fused with HD1. (**a**–**f**) GFP fluorescent images were taken from *E. coli* cells containing different combinations of split GFP constructs: (**a**) empty vector control (NC-control), (**b**) positive vector control with leucine zipper constructs (NZ-CZ), (**c**) AtCHE-AtHD1, (**d**) AaCHE-AaHD1, (**e**) AaCHE-AtHD1, (**f**) AtCHE-AaHD1. (**g**) Flow cytometer quantification of GFP expression levels as indicators for protein-protein interaction. Normalized GFP intensity was calculated based on the fluorescence intensity divided by the percentage of cells showing positive GFP signals. Error bars represent standard deviation from 3 replicates. Scale bars = 10 μm. Asterisks mark statistically significant differences between comparisons (P < 0.05).

The yeast-two hybrid data were less quantitative due to the weak interaction between CHE and HD1 (as shown with the limited growth in the triple dropout media). Therefore, we also quantified the interactions between At/AaCHE and At/AaHD1 homoeologous proteins *in vivo* using a split green fluorescent protein (GFP) complementation in a bacterial two-hybrid assay^43^. *AtCHE* and *AaCHE* cDNA were cloned and fused downstream of the N-GFP, respectively. In parallel, *AtHD1* and *AaHD1* cDNA were fused upstream of the C-GFP, respectively (see Supplementary Fig. S3). Expression of recombinant N-GFP proteins was driven by the T7 promoter in the presence of isopropyl β-D-1-thiogalactopyranoside (IPTG), while expression of the recombinant C-GFP proteins was under the control of the P_BAD_ promoter, which is inducible by L-(+)-arabinose. The expression and reassembly of the GFP were visualized using GFP fluorescence microscopy (Figs 5a–f). As expected, positive GFP signals could be seen for four protein (AtCHE, AaCHE, AtHD1 and AaHD1) combinations. GFP signals in cells containing the empty vectors and the leucine zipper constructs (NZ-CZ) were used as negative and positive controls, respectively. To normalize the difference in expression constructs, approximately 10,000 cells with positive GFP signals were counted for each protein combination in flow cytometer analysis to estimate relative levels of fluorescence intensities (Fig. 5g). Between the combination of AtCHE with AtHD1 and AaHD1, the GFP intensity was significantly higher in AtCHE-AaHD1 than in AtCHE-AtHD1, suggesting that AtCHE preferentially interacts with AaHD1. Similarly, the GFP intensity was significantly higher in AaCHE-AtHD1 than in AaCHE-AaHD1 combinations, indicating stronger binding of AaCHE with AtHD1 than with AaHD1. Therefore, the combination between proteins from different species (AaCHE-AtHD1 or AtCHE-AaHD1) has stronger interactions than the combination between the proteins from the same parent or species (AtCHE-AtHD1 or AaCHE-AaHD1). The result from the split GFP complementation approach is consistent with the result from the independent yeast two-hybrid assay (see Supplementary Fig. S4). Notably, when comparing AtCHE-AtHD1 and AaCHE-AaHD1 interactions, an opposite trend was observed between results from the yeast two-hybrid and split GFP assays. This discrepancy could result from the comparisons between two protein type variables (CHE and HD1) in each system, therefore making it difficult to interpret the results. In combination with the preferential binding of AaCHE to the *AtCCA1* promoter (Figs 3 and 4), this stronger protein interaction between the two proteins from different species suggests a biochemical basis for down-regulation of *AtCCA1* expression levels during the day in the allotetraploids^14^.

### AaCHE interacts stronger than AtCHE with TOC1

**Figure 6:**
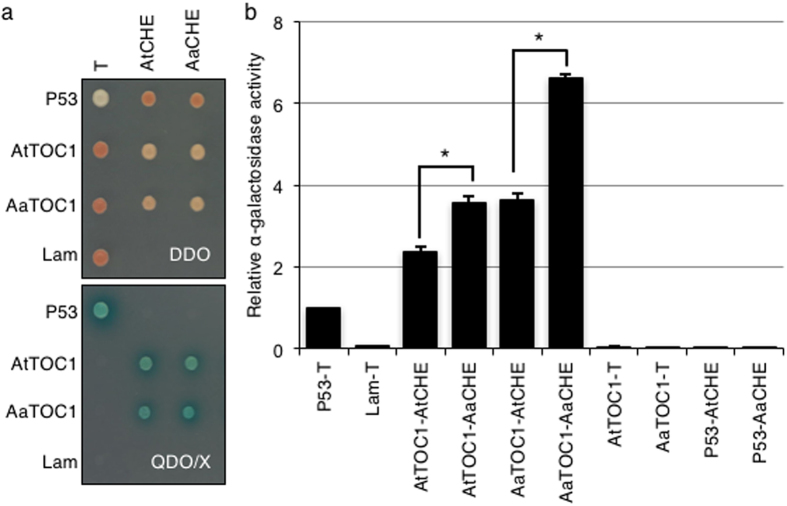
Interaction of TOC1 and CHE in yeast. (**a**) Double dropout medium (DDO) was used to select yeast strain containing the bait (AtTOC1 or AaTOC1) and prey (AtCHE or AaCHE) proteins. Qualitative protein-protein interaction was visualized using X-α-gal in quadruple dropout (QDO/X) medium. Yeast vectors expressing P53 (Gal4 DNA-BD fused with murine p53), T (Gal4 AD fused with SV40 large T-antigen), and Lam (a negative control vector with Gal4 BD fused with lamin) were used as controls in various indicated combinations. (**b**) Quantitative α-galactosidase assay for protein-protein interaction in yeast. The relative α-galactosidase activity was measured against the positive p53-T interaction. Error bars represent standard deviation from 3 biological replicates. Asterisks indicate statistically significant differences between comparisons (P < 0.05).

In the evening loop of the circadian clock, genetic studies showed that TOC1 activates *CCA1* expression through tethering CHE from the *CCA1* promoter^22^. However, TOC1 can bind to the *CCA1* promoter and repress its activities^23^. Since our data suggest a role for genetic interactions between TOC1 and CHE in regulating *CCA1* expression (Fig. 2b), we cloned the homologous AtTOC1 and AaTOC1 from the allotetraploids (see Supplementary Fig. S5) to study their interactions with CHE at biochemical levels. To achieve this, we used the yeast two-hybrid assay to test protein interactions involving AtTOC1 or AaTOC1 and AtCHE or AaCHE (Fig. 6). As expected, strong interactions between TOC1 and CHE were detected in the quadruple synthetic dropout medium (SD-Leu/-Trp/-Ade/-His) containing X-α-gal (Fig. 6a). The strength of interactions between TOC1 and CHE was quantified by a quantitative assay for X-α-gal activity^44^. When AtTOC1 was used as the bait protein, AtTOC1-AaCHE interaction was significantly stronger than the AtTOC1-AtCHE interaction. In contrast, the AaTOC1-AaCHE combination showed a stronger interaction than the AaTOC1-AtCHE combination when AaTOC1 was used as the bait protein (Fig. 6b). These data suggest that AaCHE interacts strongly with TOC1 than AtCHE regardless which combination is used and that not all homoeologous protein interactions are favored in hybrid combinations. Nevertheless, AaCHE was found to preferentially bind to the *CCA1* promoter compared to AtCHE (Fig. 3). In addition, comparing with AtTOC1, AaTOC1 showed a stronger interaction with AtCHE or AaCHE. This *in vitro* result is consistent with higher expression levels of *AtTOC1* than *AaTOC1* in the allotetraploids *in vivo*^14^. The data collectively suggest that the observed preferential protein-protein interactions between TOC1 and CHE could act in concert with the preferential binding of AaCHE to the *AtCCA1* promoter in altering the *AtCCA1* expression peaks in the allotetraploids and hybrids^14^,^25^,^45^.

## Discussion

**Figure 7:**
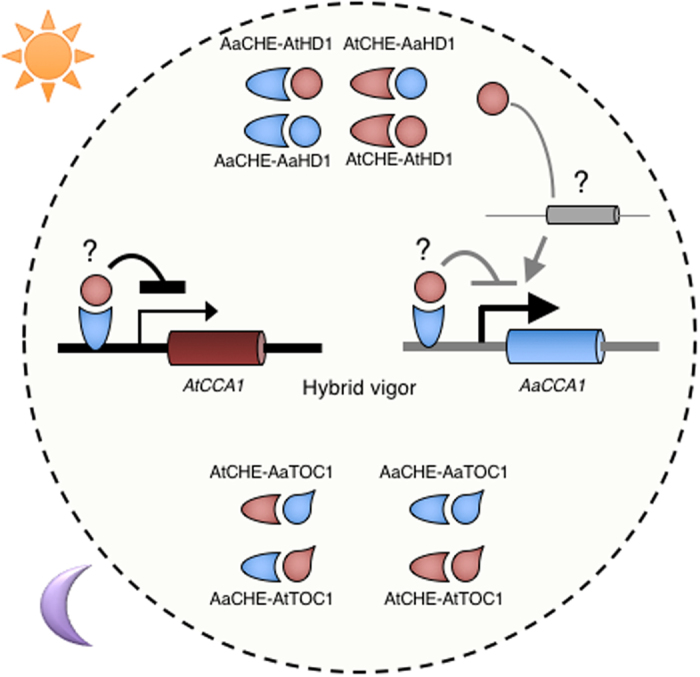
A simplified model for CHE-mediated allelic *CCA1* expression in *Arabidopsis* allotetraploids. In the morning, CHE, acts to down-regulate *CCA1* expression during the day. The down-regulation of *CCA1* by CHE could be mediated through its interaction with HD1 with a possibility of recruiting HD1 to the *CCA1* promoter. In *Arabidopsis* allotetraploids, *A. thaliana* (At) and *A. arenosa* (Aa) homoeologous proteins such as CHE and HD1 could form heterologous interactions, leading to differential binding affinity of the protein complex (e.g., AaCHE-AtHD1) to the *AtCCA1* (black) or *AaCCA1* (grey) promoter, causing more reduction on *AtCCA1* transcripts than on *AaCCA1* transcripts (light/heavy arrows indicate transcript activation levels, see Supplementary Fig. S1)^14^,^41^,^48^. In addition to the heterologous protein complex, *cis*-regulatory changes in the *AtCCA1* and *AaCCA1* promoters could also reduce *CCA1* expression levels. At night, At/AaTOC1 interacts preferentially with AaCHE, thereby, relieving *CCA1* from repression. The interactions between At/AaTOC1 and At/AaHD1 homoeologous proteins have not been tested. As a transcriptional repressor, HD1 could potentially mediate *CCA1* expression independent of CHE through other clock regulators (with a question mark) in the hybrids.

Altered gene expression and signaling pathways have been shown to potentially contribute to heterotic phenotypes in hybrids^6^,^7^,^13^. Results from this study provided a model for the fine-tuning of the circadian clock in *Arabidopsis* allotetraploids. The heterologous protein interaction model could provide a biochemical explanation for biased expression of homoeologs in interspecific hybrids or allotetraploids (Fig. 7). Within the central oscillator of the clock, CCA1, a MYB-related transcription factor, binds to the evening element of its target genes^14^,^39^. In *A. thaliana, CCA1* expression is mediated by both CHE and TOC1^17^,^21^. In the allotetraploids, AaCHE preferentially binds to the *AtCCA1* promoter, thereby repressing its expression during the day. Such preference of protein-DNA interactions could lead to the down-regulation of the *AtCCA1* homoeolog in the allotetraploids^14^. In this study, both the yeast two-hybrid assay and the bacterial assay with split GFP complementation confirmed the interaction of CHE and HD1. Since HD1 is a general transcriptional regulator^36^,^42^, the repression of *CCA1* alleles by CHE could be mediated through preferential interactions between AtHD1 and CHE. However, the recruitment of HD1 to the *CCA1* promoter through its interaction with CHE remains to be determined. As a general transcriptional repressor, HD1 could be targeted to other clock regulators and output genes in the circadian-mediated transcriptional network, which in turn, indirectly contributes to *CCA1* expression variation during the day. The preferential interaction between AaCHE and TOC1 could also contribute to the allelic expression differences of *CCA1* homoeologs in the allotetraploids. As a result, *CCA1* expression bias could affect other downstream targets of the clock, contributing to altered metabolic and signaling pathways in the hybrids. Although the results from *in vitro* studies should be further tested to confirm the interaction of CHE and HD1 homoeologs *in planta* using alternative approaches such as bimolecular fluorescence complementation^46^, the presence of both transgene and endogenous duplicate genes may complicate data interpretation. In addition, generating and characterizing transgenic allotetraploid plants is not a trivial task.

In the biological network regulated by the circadian clock, hybrids or allotetraploids induce allelic expression changes of the regulatory genes such as *CCA1*. This allelic expression variation are associated with the parent-of-origin effect^34^ and histone modifications^14^. At the protein level, transcription factors such as CHE from maternal (red) and paternal (blue) alleles in the hybrids or allotetraploids could form homo- or heterodimers (Fig. 7), which change biochemical functions including inhibitory effects^12^ or binding affinities to the promoters of downstream genes in a biological pathway, leading to heterozygote advantages in the hybrids^6^. Given the complexity of circadian clock feedback loops, the heterologous protein-protein and/or protein-DNA interactions could alter the overall clock-mediated transcriptome in the allotetraploids, providing a potential for acquiring novel adaptive traits in the hybrids. At the systems level, overall protein stability and metabolism are predicted to play a critical role in heterosis^13^. In particular, one can predict that the interactions between heterologous subunits of transcription regulators such as the circadian-clock proteins enhance the strength to regulate output traits directly relevant to the development and evolution of hybrids and polyploids in plants and animals. Therefore, heterologous protein-protein and protein-DNA interactions could be generalized to provide a biochemical basis for biased expression of circadian clock genes that are associated with biomass heterosis^14^.

## Methods

### Plant materials and growth conditions

*A. thaliana* T-DNA mutants, *che1* (SALK_143403) and *hda19* (SALK_139445) were obtained from the Arabidopsis Biological Resource Center (ABRC). Sources of other plant materials include *athd1*-*t1*^31^, *toc1*-*101*^47^, and ColC (*ProCCA1:LUC* in Col-0 ecoptype)^34^. All plants were grown at 22 °C under a 16/8-h light/dark cycle.

### Cloning of *A. thaliana* (At) and *A. arenosa* (Aa) clock genes from the allotetraploids

Since AtCHE (*At5g08330*) contains no intron, therefore, genomic DNA encoding the homoeologous AtCHE and AaCHE were amplified from genomic DNA of the resynthesized *Arabidopsis* allotetraploid (Allo) and cloned into pGEM-T vector (Promega, Madison, WI, USA). The resulting clones, pGEM-T/AtCHE and pGEM-T/AaCHE were verified by sequencing and used for other plasmid constructions in this study. For homoeologous HD1 and TOC1 cDNA, they were amplified from cDNA prepared from Allo and cloned into pGEM-T. The resulting clones, pGEM-T/AtHD1, pGEM-T/AaHD1, pGEM-T/AtTOC1 and pGEM-T/AaTOC1 were verified by sequencing and used for cloning of plasmids for the yeast two-hybrid assay. All cloning were done using Phusion high-fidelity DNA polymerase (New England Biolabs, Ipswich, MA, USA) following manufacturer’s instructions. Primers for cloning of the At and Aa clock genes were listed in Supplementary Table S1.

### Bacterial expression and purification of recombinant CHE

CHE cDNA inserts from pGEM-T/AtCHE and pGEM-T/AaCHE, respectively, were isolated using *Eco*RI and *Sal*I restriction digestion and cloned into the pMalC2 vector (New England Biolabs, Ipswich, MA, USA). The homologous CHE was fused downstream of the Maltose binding protein and a TEV protease cleave site, yielding pMalC2/AtCHE and pMalC2/AaCHE, respectively. For bacterial expression of the recombinant proteins (MBP-rAtCHE or MBP-rAaCHE), corresponding plasmid was transformed into BL21-CodonPlus(DE3)-RIL competent cells (Stratagene, Santa Clara, CA, USA). Expression of the recombinant proteins was induced by subculturing 1 mL starter culture (OD_600_ = 0.5) into 100 mL LB media containing 1 mM isopropyl β-D-1-thiogalactopyranoside (IPTG) and shaking at 37 °C for 2.5 hours. For protein purification, bacterial cells were harvested from 40 mL induced culture ‘resuspended in 1 mL column buffer (20 mM Tris HCl pH7.4, 200 mM NaCl, 1 mM EDTA) containing 1 mg/mL lysozyme, and incubated on ice for 30 minutes. Then the bacterial cells were lysed by sonication and supernatant from the lysed cultures were collected for subsequent column purified using amylose resin (New England Biolabs, Ipswich, MA, USA) and finally eluted with 1 mL maltose (10 mM). The eluted proteins were then quantified using Bradford assay (BioRad) and 2 pmol/μL purified MBP, MBP-rAtCHE and MBP-rAaCHE were prepared as protein working stocks for subsequence electrophoretic mobility shift assay. The quality of purified proteins was verified using 10% SDS-PAGE.

### Split-GFP reassembly constructs

The split-GFP reassembly vectors (pET11a-link-NGFP and pMRBAD-link-CGFP) and the positive control plasmids (pET11a-Z-NGFP and pMRBAD-Z-CGFP) were kindly provided by Lynne Regan from Yale University. Target cDNAs (AtCHE, AaCHE, AtHD1 and AaHD1) were amplified from the corresponding cloned cDNA from the allotetraploids. Restriction enzyme sites flanking the cDNA were introduced in the primers for cloning into the split-GFP vectors (see Supplementary Table S1). *AtCHE* and *AaCHE* were cloned into pET11a-link-NGFP for bacteria expression of recombinant NGFP:AtCHE and NGFP:AaCHE, respectively. *AtHD1* and *AaHD1* were cloned into pMRBAD-link-CGFP for bacterial expression of AtHD1:CGFP and AaHD1:CGFP, respectively.

### qRT-PCR

For gene expression analyses, mature leaves from 3–4 weeks old plants were harvested. Total leaf RNAs were prepared using plant RNA reagent (Life technologies, Carlsbad, CA, USA) according to manufacturer’s instructions. cDNA synthesis was performed using Omniscript reverse transcription kit (Qiagen, Venlo, Limburg, Netherlands). Quantitative RT-PCR analysis was performed using Faststart universal SYBR green master (Rox) (Roche, Basel, Switzerland) in an ABI7500 machine (Applied Biosystems, Waltham, MA, USA).

### Bioluminescence assays and data analysis

Luciferase reporter line containing the *ProCCA1:LUC* construct^34^ was crossed with various clock mutants (*che, hd1* or *toc1*) and homozygous transgenic lines in various single or double mutants (*che*/*hd1* or *che*/*toc1*) were selected for luciferase expression assay using a TopCount NXT luminometer and scintillation count (Perkin-Elmer, Waltham, MA, USA) and the means ± SE luciferase activity was calculated following the procedures described in ref. 34.

### Electrophoretic mobility shift assay (EMSA)

For EMSA, the homologous *CCA1* promoter (*CCA1p*) containing the CHE binding site (CHE BS) were PCR amplified using cloned *AtCCA1p* and *AaCCA1p* fragments from the allotetraploids (see Supplementary Table S2). The resulting PCR products, *AtCCA1p* (133 bp) and *AaCCA1p* (134 bp), were then gel purified. Equal molar (3 pmol) of the purified PCR products were then end-labeled with T4 polynucleotide kinase in the presence of 32^P^-γATP (3000 Ci/mmol, 10 mCi/mL). End-labeled probes were then purified using Sephadex G-50 column with STE buffer (10 mM Tris HCl pH8, 100 mM NaCl and 1 mM EDTA). For EMSA, 10 nM (~15 fmol) DNA were used for binding reaction in buffer containing 25 mM HEPES pH7.5, 2.5 mM DTT, 75 mM KCl, 10% glycerol and 1.25 ng poly-dIdC. Various amount of the purified MBP, MBP-rAtCHE, and MBP-rAaCHE were then used for the binding reactions. The binding reactions were incubated on ice for 20 minutes and then revolved with 5% native polyacrylamide gel electrophoresis. After electrophoresis, the gel was dried on a 3 M filter paper and exposed using X-ray film. For competition assays, 1x, 5x, 25x, and 125x unlabeled cold probes were included in the binding reactions. Densitometric intensities of the shifted DNA probes were quantified using ImageJ software (National Institutes of Health).

### Protein expression for GFP fragments reassembly

A pair of GFP reassembly constructs was co-transformed into Rossetta2 (DE3) competent cells. For a negative control, both pET11a-link-NGFP and pMRBAD-link-CGFP constructs were used. For a positive control, pET11a-Z-NGFP and pMRBAD-Z-CGFP expressing the leucine zipper reassembly constructs were used^43^. The interacting pairs of co-transformation were 1) NGFP:AtCHE and AtHD1:CGFP; 2) NGFP:AaCHE and AaHD1:CGFP; 3) NGFP:AtCHE and AaHD1:CGFP; and 4) NGFP:AaCHE and AtHD1:CGFP, respectively. For bacterial expression, 10 ng of each Miniprep purified DNA in 5 μL water were co-transformed into 50 μL Rossetta2 (DE3) competent cells. The double transformants were selected on LB agar plates containing 100 μg/mL ampicillin, 35 μg/mL kanamycin and 40 μg/mL chloramphenicol. A single colony was picked from the triple-selection agar plate for inoculation in 5 mL LB containing ampicillin, kanamycin and chloramphenicol cultured overnight at 37 °C with constant shaking. For recombinant protein induction and GFP fragment reassembly, fresh overnight culture was diluted to 1:1000 and 5 μL of the diluted culture were plated onto a reassembly plate containing 10 μM IPTG, 0.2% arabinose and the antibiotics, ampicillin, kanamycin and chloramphenicol. The plate was divided into sections based on number of samples and controls. Each pair of treatments was repeated from 5 independent cultures and reassembly plating. The plates were incubated at 30 °C overnight followed by additional 2 days at 20 °C.

### GFP fluorescence microscopy

Bacteria containing the GFP reassembly constructs were resuspended in 0.5 mL buffer (25 mM Tris-HCl, pH8.0, 10% glycerol) and the OD_600_ of the resuspended cells were adjusted to 2. For fluorescence microscopy, 20 μL of each resuspended cells were transferred onto a Superfrost/Plus microscope slide (Fisher Scientific, Hampton, New Hampshire, USA) and covered with 24 × 40 mm micro cover glass (VWR, Radnor, Pennsylvania, USA). Edges of the cover glasses were sealed with nail polish. Live cell images were taken on a Zeiss Axiovert 200 M inverted microscope under brightfield transmission and FITC fluorescence filter using 20× objective. Three representative fields from each slide were taken.

### GFP quantification by FACSCalibur

Fresh overnight bacterial cultures were diluted to 1:10000 and 100 μL of the diluted cultures were inoculated into 5 mL LB media supplemented with 100 μg/mL ampicillin, 35 μg/mL kanamycin and 40 μg/mL chloramphenical and incubated at 37 °C with constant shaking. When OD_600_ reached 0.5, IPTG (15 μM) and arabinose (0.2%) were added into each 5 mL culture for recombinant proteins induction. The cultures were incubated at 20 °C incubator for 2 days. The cells were centrifuged at 2000 g for 15 minutes at 4 °C and washed twice with 25 mM Tris-HCl, pH8.0, 10% glycerol buffer. The harvested cells were resuspended in 25 mMTris-HCl buffer with OD_600_ = 1. FACSCalibur samples were prepared by diluting each cell culture with 25 mM Tris-HCl (pH8.0) buffer, resulting in a concentration of ~ 2 × 10^6^ cells/mL in a 5 mL PolyStyrene Tube w/Filter Cap (Fisher Scientific, Hampton, New Hampshire, USA). FACSCalibur flow cytometer (BD Biosciences, San Jose, CA, USA) analysis was used to evaluate relative levels of fluorescence intensity in bacterial cells co-expressing the GFP reassembly constructs pair (NGFP:CHE and HD1:CGFP). Samples were run under low pressure using the FL2 detector set at 480 nm. A total of ~10,000 events were counted for each sample, and three biological replicates were performed for each treatment. Flow-cytometry data were collected and analyzed using CellQuestPro software (BD Biosciences, San Jose, CA, USA). A scatter plot was generated from the collected data. Cells with positive GFP signals were then identified and gated. Relative proportion of GFP within these gated peak regions (FL2-A) were calculated and tested for statistical significance.

### Yeast two-hybrid assays

For the bait plasmids construction, homologous CHE and TOC1 inserts were cut from the corresponding clones in the pGEM-T plasmids through *Eco*RI and *Sal*I restriction sites and cloned into the pGBKT7-BD respectively. For the prey plasmids construction, homologous CHE were cloned through *Eco*RI and *Sac*I restriction sites; and HD1 were cloned through *Xho*I and *Xma*I restriction sites, into the pGADT7-AD respectively. For yeast two-hybrid, a matchmaker gold yeast two-hybrid system and a yeastmaker yeast transformation system 2 (Clontech, Mountain View, CA, USA) were used. Yeast two-hybrid screens were conducted according to manufacturers’ instructions. For yeast transformation, yeast strain Y2H gold was used for bait plasmid transformation while the yeast strain Y187 was used for prey plasmid transformation. For qualitative assessment of interaction between the selected target proteins pair, transformed yeast colony (2–3 mm) containing the target baits or prey plasmids were mixed and incubated at 30 °C with shaking at 200 rpm for overnight. The successful mated yeast strains were selected on double dropout media without Leu and Trp (DDO). Mated diploid yeast strains were also selected and screen on double dropout media containing X-α-gal (DDO/X), Triple dropout media (without Leu, Trp and His) containing X-α-gal (TDO/X) or quadruple dropout media (without Leu, Trp, His and Ade) containing X-α-gal (QDO/X). The selected diploid hybrid yeast containing both the bait and prey plasmids in DDO liquid media and shook (350 rpm) overnight at 30 °C. Then, 0.6 × 10^7^ cells (OD_600_ = 0.6) were harvested, resuspended in 3 mL DDO media and shook (250 rpm) at 29 °C for 16 hours. For quantitation of reporter expression (α-galactosidase) resulted from positive protein-protein interaction, a quantitative p-nitrophenyl-a-D-galactopyranoside (PNG) assay was used^44^. To perform the assay, the subcultured cells were pelleted at 13,000 rpm for 1 minute and 200 μL supernatant was transferred to a new tube containing 600 μL assay buffer (33 mM PNG, 0.33 M sodium acetate pH4.5). The reaction was incubated at 29 °C for 24 hours, stopped by adding 200 μL 2 M Na_2_CO_3_ and OD_410_ of the stopped reaction was measured. For quantification, the α-galactosidase activity was estimated as the change of OD_410_/minute/cell. For each interacting proteins partner, the relative α-galactosidase activity was measure against the positive P53-T positive control.

### Accession numbers

Sequence data from this article can be found in the GenBank/EMBL databases under the following accession numbers: *At2g46830 (AtCCA1*), *At5g08330 (AtCHE*), *At5g61380 (AtTOC1*), *At4g38130 (AtHD1*), *At1g07930 (EF1α*).

## Additional Information

**How to cite this article:** Ng, D. W-K. *et al*. Heterologous protein-DNA interactions lead to biased allelic expression of circadian clock genes in interspecific hybrids. *Sci. Rep.* **7**, 45087; doi: 10.1038/srep45087 (2017).

**Publisher's note:** Springer Nature remains neutral with regard to jurisdictional claims in published maps and institutional affiliations.

## Supplementary Material

Supplementary Information
