# Supplementary material for: Heterologous protein-DNA interactions lead to biased allelic expression of circadian clock genes in interspecific hybrids
Source: Sci Rep. 2017 Mar 27;7:45087. doi: 10.1038/srep45087 (PMC5366859; doi:10.1038/srep45087)
Supplement: Supplementary Information [file srep45087-s1.pdf]

**Supplementary information**

# Heterologous protein-DNA interactions contribute to biased allelic expression of circadian clock genes in interspecific hybrids

Danny W-K Ng, Helen H. Y. Chen, and Z. Jeffrey Chen

The following materials are available in the online version of this article.

**Supplementary Figures**

**Supplementary Figure S1:** Expression of *CCA1*, *CHE* and *HD1* in the allotetraploids [1]

**Supplementary Figure S2:** ClustalW alignment of homoeologous AtCHE and AaCHE proteins.

**Supplementary Figure S3:** ClustalW alignment of homoeologous AtHD1 and AaHD1 proteins.

**Supplementary Figure S4:** Interaction of CHE and HD1 in yeast.

**Supplementary Figure S5:** ClustalW alignment of homoeologous At and Aa TOC1.

**Supplementary Tables**

**Supplementary Table S1**: Oligonucleotide primers for cloning [2].

**Supplementary Table S2:** Oligonucleotide primers used in gene expression analyses and EMSA assay.

**References**

1. Shi, X., Zhang, C., Ko, D.K. & Chen, Z.J. Genome-Wide Dosage-Dependent and -Independent Regulation Contributes to Gene Expression and Evolutionary Novelty in Plant Polyploids. *Mol Biol Evol* **32**, 2351-66 (2015).
2. Ng, D.W. *et al.* A Role for CHH Methylation in the Parent-of-Origin Effect on Altered Circadian Rhythms and Biomass Heterosis in *Arabidopsis* Intraspecific Hybrids. *Plant Cell* **26**, 2430-2440 (2014).

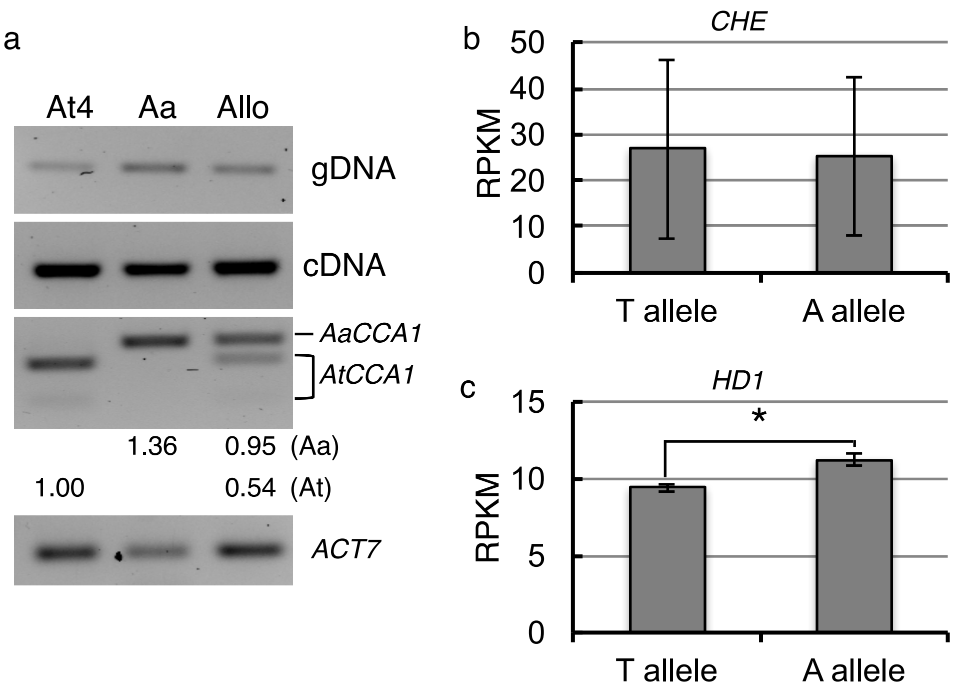


**Supplementary Figure S1: Expression of *CCA1*, *CHE* and *HD1* in the allotetraploids.** (**a**) Relative expression of the *CCA1* alleles from the *Arabidopsis thaliana* (At) and the *Arabidopsis arenosa* (Aa) genomes in the allotetraploids (Allo). Semi-quantitative RT-PCR and CAPS analyses were used to detect the relative alleles expression in the mature leaves. Genomic DNA (gDNA) were used as a positive control. cDNA were digested with AvaII to determine the relative alleles (*AaCCA1* and *AtCCA1*) expression in Allo. *ACT7* was used as a control. The relative At/ Aa allele intensities were determined by ImageJ. RPKM value for T allele (At4, *Arabidopsis thaliana*) and A allele (Aa, *Arabidopsis arenosa*) expression in *Arabidopsis* allotetraploids (Allo) at ZT6. (**b**) *CHE* and (**c**) *HD1*. Error bars indicated standard deviation from 3 replicates. Data for b and c are derived from a previous report by Shi et al. (2015) [1].

```
AtCHE MADNDGAVSNGIIVEQTSNKGPLNAVKKPPSKDRHSKVDGRGRRIRMPIICAARVFQLTR 178
AaCHE         T  V I                                               178

AtCHE ELGHKSDGQTIEWLLRQAEPSIIAATGTGTTPASFSTASLSTSSPFTLGKRVVRAEEGES 358
AaCHE                                                              358

AtCHE GGGGGGG-LTVGHTMGTSLMGGGGSGGFWAVPARPDFGQVWSFATGAPPEMVFAQQQQPA 535
AaCHE        G                                    A                538

AtCHE TLFVRHQQQQQASAAAAAAMGEASAARVGNYLPGHHLNLLASLSGGANGSGRREDDHEPR 715
AaCHE                                                              718
```

**Supplementary Figure S2: ClustalW alignment of homoeologous AtCHE and AaCHE proteins**. Consensus protein sequences between At and Aa are in black. Amino acid residues differ between the two homoeologs are highlighted in red.

```
AtHD1 MDTGGNSLASGPDGVKRKVCYFYDPEVGNYYYGQGHPMKPHRIRMTHALLAHYGLLQHMQ 60
AaHD1                                                              60

AtHD1 VLKPFPARDRDLCRFHADDYVSFLRSITPETQQDQIRQLKRFNVGEDCPVFDGLYSFCQT 120
AaHD1                                                              120

AtHD1 YAGGSVGGSVKLNHGLCDIAINWAGGLHHAKKCEASGFCYVNDIVLAILELLKQHERVLY 180
AaHD1                                                              180

AtHD1 VDIDIHHGDGVEEAFYATDRVMTVSFHKFGDYFPGTGHIQDIGYGSGKYYSLNVPLDDGI 240
AaHD1                                              N               240

AtHD1 DDESYHLLFKPIMGKVMEIFRPGAVVLQCGADSLSGDRLGCFNLSIKGHAECVKFMRSFN 300
AaHD1                   V                                          300

AtHD1 VPLLLLGGGGYTIRNVARCWCYETGVALGVEVEDKMPEHEYYEYFGPDYTLHVAPSNMEN 360
AaHD1                                                              360

AtHD1 KNSRQMLEEIRNDLLHNLSKLQHAPSVPFQERPPDTETPEVDEDQEDGDKRWDPDSDMDV 420
AaHD1                                      A                       420

AtHD1 DDDRKPIPSRVKREAVEPDTKDK---DGLKGIMERGKGCEVEVDESGSTKVK         470
AaHD1                        FVQ     V         G      S            473
```

**Supplementary Figure S3: ClustalW alignment of homoeologous AtHD1 and AaHD1 proteins.** Consensus protein sequences between At and Aa are in black. Amino acid residues differ between the two homoeologs are highlighted in red.

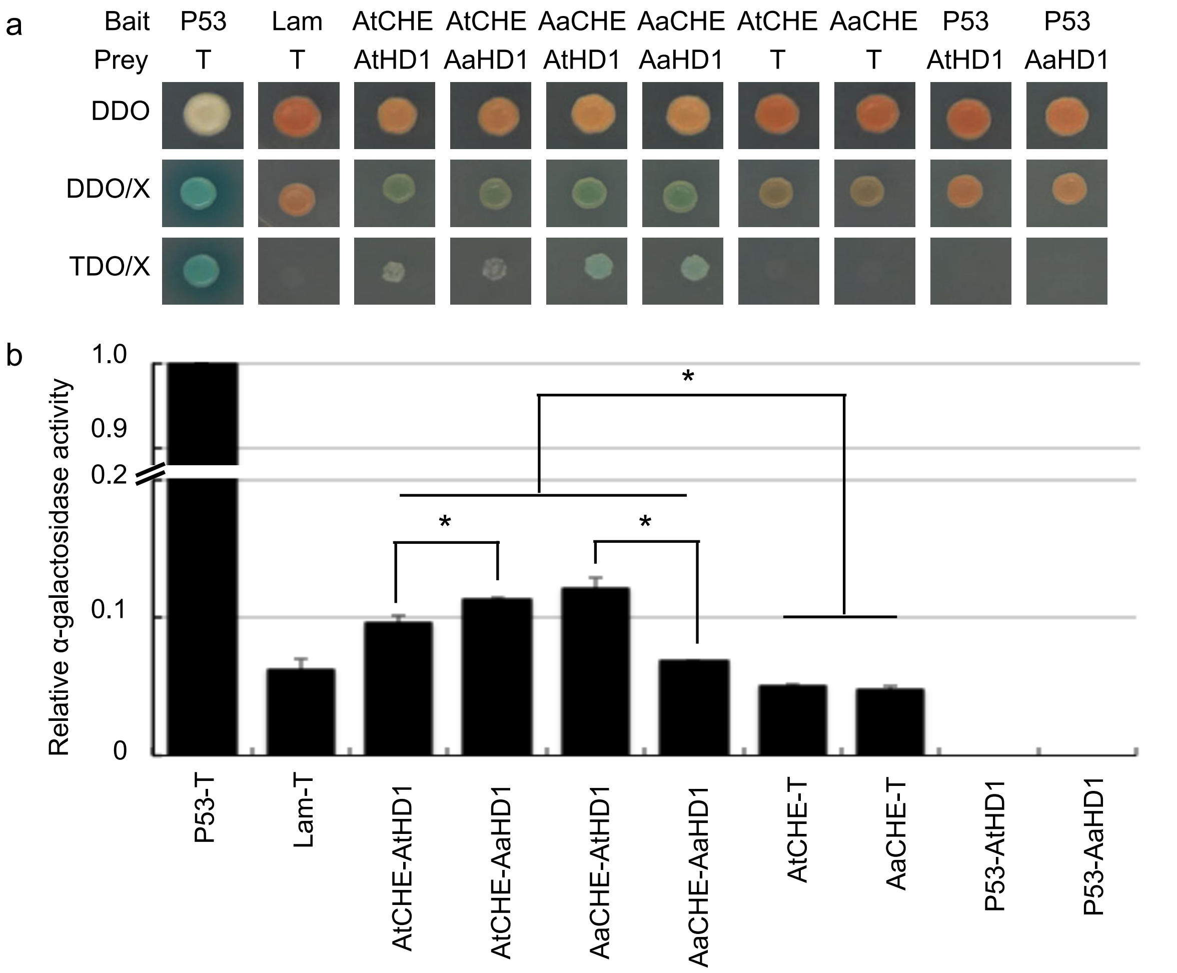


**Supplementary Figure S4: Interaction of CHE and HD1 in yeast.** (**a**) Double dropout medium (DDO) was used to select yeast strain containing the bait (ATCHE or AaCHE) and prey (AtHD1 or AaHD1) proteins. Qualitative protein-protein interaction was visualized using X-α-gal in double dropout (DDO/X) and triple dropout (TDO/X) media. Yeast vectors expressing P53 (Gal4 DNA-BD fused with murine p53), T (Gal4 AD fused with SV40 large T-antigen), and Lam (a negative control vector with Gal4 BD fused with lamin) were used as controls in various indicated combinations. (**b**) Quantitative α-galactosidase assay for protein-protein interaction in yeast. The relative α-galactosidase activity was measured against the positive p53-T interaction. Error bars represent standard deviation from 3 replicates. Asterisks mark statistically significant differences between comparisons ($P < 0.05$).

```
AtTOC1 MDLNGECKGGDGFIDRSRVRILLCDNDSTSLGEVFTLLSECSYQVTAVKSARQVIDALNA 60
AaTOC1                             K                 S              60

AtTOC1 EGPDIDIILAEIDLPMAKGMKMLRYITRDKDLRRIPVIMMSRQDEVPVVVKCLKLGAADY 120
AaTOC1                                                              120

AtTOC1 LVKPLRTNELLNLWTHMWRRRRMLGLAEKNMLSYDFDLVGSDQSDPNTNSTNLFSDDTDD 180
AaTOC1                                           P                  180

AtTOC1 RSLRSTNPQRGNLSHQENEWSVATA----PVHARDGGLGADGTATSSLAVTAIEPPLDHL 236
AaTOC1                          TAPA AR G                           240

AtTOC1 AGSHHEPMKRNSNPAQFSSAPKKSRLKIGESSAFFTYVKSTVLRTNGQDPPLVDGNGSLH 296
AaTOC1    Y         L                                       N       300

AtTOC1 LHRGLAEKFQVVASEGINNTKQARRATPKSTVLRTNGQDPPLVNGNGSHHLHRGAAEKFQ 356
AaTOC1           A             GT      F      S        LP    L      360

AtTOC1 VVASEGINNTKQAHRSRGTEQYHSQGETLQNGASYPHSLERSRTLPTSMESHGRNYQEGN 416
AaTOC1 A            RG  EN K      N        Y     C                  420

AtTOC1 MNIPQVAMNRSKDSSQVDGSGFSAPNAYPYYMHGVMNQVMMQSAAMMPQYGHQIPHCQPN 476
AaTOC1   T                                                          480

AtTOC1 HPNGMTGYPYYHHPMNTSLQHSQMSLQNGQMSMVHHSWSPAGNPPSNEVRVNKLDRREEA 536
AaTOC1                                                              540

AtTOC1 LLKFRRKRNQRCFDKKIRYVNRKRLAERRPRVKGQFVRKMNGVNVDLNGQPDSADYDDEE 596
AaTOC1                                                              600

AtTOC1 EEEEEEEEENRDSSPQDDALGT                                       619
AaTOC1                                                              623
```

**Supplementary Figure S5: ClustalW alignment of homoeologous At and Aa TOC1.** Consensus protein sequences between At and Aa TOC1 are in black. Amino acid residues differ between the two homoeologs are highlighted in red.

## Supplementary Tables

### Supplementary Table S1: Oligonucleotide primers for cloning.

| Name | Sequence (5' - 3')* | Target(s) |
|---|---|---|
| EcoRI-CHE-F[1,2,3,4] | gagaattcATGGCCGACAACGACGGA | *At/Aa CHE* |
| SalI-CHE-R[1,2,3,4] | ctatgtcgacTCAACGTGGTTCGTGGTC | *At/Aa CHE* |
| EcoRI-XhoI-AtTOC1-F[1,3] | gaattcctcgagATGGATTTGAACGGTGAG | *AtTOC1* |
| SalI-AtTOC1-R[1,3] | gtcgacTCAAGTTCCCAAAGCATCATC | *AtTOC1* |
| EcoRI-XhoI-AaTOC1F[1,3] | gaattcctcgagATGGATTTGAACGGTGAGTG | *AaTOC1* |
| SalI-AaTOC1-R[1,3] | gtcgacTAGGTATGAACACAACATGGTTTTCAG | *AaTOC1* |
| XmaI-AtHD1-F[1,4] | cccgggaGATACTGGCGGCAAT | *At/Aa HD1* |
| XhoI-AtHD1-R[1,4] | ctcgagTCATTTTACCTTAGTGCTTCCACTC | *AtHD1* |
| XhoI-AaHD1-R[1,4] | ctcgagTCATTTTACCTTACTGCTTCCACTC | *AaHD1* |
| XhoI-CHE-F[5] | aataatctcgagcgaattcATGGCCGACAAC | *At/Aa CHE* |
| BamHI-CHE-R[5] | aataatggatccttatcAACGTGGTTCGTGGTC | *At/Aa CHE* |
| NcoI-HD-F[5] | aataatccatggctccgggaGATACTGGCGGCAAT | *At/Aa HD1* |
| AatII-AtHD-R[5] | aataagacgtcccttaTGTTTTAGGAGGAAACGC | *AtHD1* |
| AatII-AaHD-R[5] | aataagacgtcccttaTTTTACCTTACTGCTTCCACT | *AaHD1* |

*Small case letters indicate the sequence with added restriction enzyme sites in the gene-specific primer
[1]Oligonucleotide primers amplifying At and Aa homoeologs from allotetraploids
[2]Oligonucleotide primers for cloning of expression constructs in bacteria
[3]Oligonucleotide primers for cloning target bait construct in yeast two-hybrid assay
[4]Oligonucleotide primers for cloning target prey construct in yeart two-hybrid assay
[5]Oligonucleotide primers for cloning of GFP re-assembly constructs
Aa primer sequences were designed based on available Aa contig sequences (Shi et al. 2015) [2]

**Supplementary Table S2: Oligonucleotide primers used in gene expression analyses and EMSA assay.**

| Name | Sequence (5' - 3') | Target(s) |
|---|---|---|
| CHE-F[1] | TAATGGGTGGTGGTGGTTCTG | *AtCHE* |
| CHE-R[1] | GCAAAGCTCCAGACTTGTCC | *AtCHE* |
| HDA19-F[1] | GTCTGGTGATAGGTTGGGGTGCTTTA | *AtHD1* |
| HDA19-R[1] | CAACTTCAACTCCAAGTGCAACTCCA | *AtHD1* |
| 1319EF1a-F[1] | GACATGAGGCAGACTGTTGCA | *EF1-α* (*At1g07930*) |
| 1381EF1a-R[1] | CCGGTTGGGTCCTTCTTGT | *EF1-α* (*At1g07930*) |
| CCA1-F[2] | CTCTGCTGCATCCTCCATGAATAAAAGT | *At/Aa CCA1* |
| CCA1-R[2] | TCGGATAAGTCTGAGGTCCTTGCTCATT | *At/Aa CCA1* |
| 315CCA1p-F[3] | GTCTAGATTCCCGGGTCCACTG | *At/Aa CCA1* probe |
| 183CCAp-R[3] | GAAAGGTTAAAAAGGTCAATTCGT | *At/Aa CCA1* probe |

[1]Oligonucleotide primers for qPCR
[2]Oligonucleotide primers for PCR
[3]Oligonucleotide primers for EMSA probes
